# Comparison between Conventional Decalcification and a Microwave-Assisted Method in Bone Tissue Affected with Mycetoma

**DOI:** 10.1155/2020/6561980

**Published:** 2020-08-01

**Authors:** Magdi Mansour Salih

**Affiliations:** Histopathology and Cytology, College of Applied Medical Sciences, Clinical Laboratory Sciences Department, Taif University, Taif, Saudi Arabia

## Abstract

Mycetoma is a lifelong granulomatous disease of subcutaneous tissues and bones. Histopathology is a substantiated indicative method based on the assumption of a definitive diagnosis of mycetoma. It requires efficient processing of tissues including bone decalcification. The decalcification process must ensure complete removal of calcium and also a proper preservation of tissue and microorganisms' staining ability. *Objectives*. To compare the conventional method used in decalcification with the microwave method using different decalcification solutions. Different characteristics were tested, including the speed of decalcification and morphological and fungal preservation in bone tissue affected with mycetoma. *Materials and Methods*. Three decalcification solutions were employed to remove calcium from 50 bone tissue samples affected with mycetoma, including 10% neutral buffered EDTA (pH 7.4), 5% nitric acid, and 5% hydrochloric acid. Conventional and microwave methods were used. Haematoxylin-eosin (HE) stain, Gridley's stain, and Grocott hexamine-silver stain were employed to evaluate the bone and fungi morphologies. *Results*. The decalcification time of the conventional method compared with the microwave method with 10% EDTA (pH 7.4) took 120 hours and 29 hours, while 5% hydrochloric acid and 5% nitric acid took 8 hours and 3 hours, separately. Also, 10% EDTA is the best decalcifying agent for HE staining and fungal stains. 5% hydrochloric acid and 5% nitric acid can be used for fungal staining. *Conclusion*. The current study investigated the effects of different decalcifying agents as well as two decalcification procedures on the preservation of the bone structure and fungal staining, which will help to develop suitable protocols for the analyses of the bone tissue affected with mycetoma infection.

## 1. Introduction

Mycetoma is a lifelong granulomatous, gradually deleterious epidemic disease of the skin and subcutaneous tissues that can progress to deeper structures like muscles and bones and lead to extensive destruction, mainly of the feet, requiring wide local surgical excisions or limb amputation [1]. Mycetoma is defined by the triumvirate of expansion, exhausting sinuses, and existence of colonial grains in the inflammatory exudates [2, 3]. Infection is classified as eumycetoma (fungal infection) or actinomycetoma (bacterial infection) [4]. It is broadly a condition of tropical and semitropical regions, noticeably Sudan [5]. *Madurella mycetomatis* crop grains are huge, ranging from 0.5 to 3 mm, and appear rounded, oval, or trilobed. They consist of crisscross hyphae ingrained in interstitial brownish cement, consisting of melanin-like, black-brown pigment [6, 7]. Histopathology is a fast indicative method as well as advantageous process on the assumption of a definitive diagnosis of mycetoma disease and includes a report that describes the morphological presentation of the causative agents. The causative agent could still be isolated from the bone tissue involved [5]. Decalcification is a fundamental step that is commonly achieved for histopathological examination of bones [8]. The minerals in bones consist of calcium and phosphorus, and insoluble salts comprise more than sixty percent of bone tissue [9]. These minerals provide bone hardness and are the cause of difficulties during tissue cutting using rotary microtomes [10]. Such tissues must be treated to extract calcium phosphate by a procedure known as decalcification, through making the tissue sufficiently delicate to be cut by the microtome. Decalcification is accomplished by acids that form soluble calcium salts or chelating agents that bind to calcium ions. The current conventional decalcifying methods are characterized by laborious processes and the persistent failure of tissues' staining reaction [11]. In the conventional method of decalcification, bone tissues are placed in a decalcifying fluid at room temperature with changes of the solution at regular intervals till the end point is reached. Microwave decalcification is an innovative technique compared with the conventional method [12]. In this process, solid tissues are placed in the decalcifying solution in a microwave oven for periodic durations with usual shifts of the decalcifying fluids till the end point is attained. Microwave radiation has been conducted to accelerate the procedure of decalcification approximately from days to hours [13]. The aim of the present study was to compare the conventional method used in decalcification with the modified microwave method using different decalcification solutions. Different characteristics were tested, including the speed of decalcification and morphological and fungal preservation in bone tissue affected with *Madurella mycetomatis* infection.

## 2. Materials and Methods

This is an experimental descriptive study aimed to compare the conventional decalcifying procedure with microwave-enhanced decalcification with respect to tissue morphology affected by mycetoma infection using haematoxylin and eosin, Gridley, and Grocott hexamine-silver stains for complete identification of the *Madurella mycetomatis* causal organisms. This study was conducted at the Mycetoma Research Center in Soba University Hospital and the Faculty of Medical Laboratory Science, University of Al Neelain. Written informed consent was obtained from all patients. Fifty amputated limbs affected with mycetoma were collected. Bone biopsies were cut using a suitable saw into 5 mm-thick pieces and then fixed in 10% formal saline for 48 hours. They were washed under running tap water for 30 minutes to remove the fixative.

### 2.1. Conventional Decalcification Procedure

Three pieces of 5 mm-thick sections of bone biopsies were submerged in three 250 ml Pyrex Squat Beakers each containing 100 ml of 5% aqueous hydrochloric acid (HCl), 5% aqueous nitric acid (HNO_3_), and 10% ethylenediaminetetraacetic acid (EDTA) placed at room temperature (average 28°C), see Table 1. The end point of decalcification was checked using the calcium oxalate method [14] for the two acid decalcifiers (5% HNO_3_ and 5% HCl) after a two-hour interval as follows: 5 ml of the used decalcifying fluid was taken and placed in a test tube, then litmus paper was added, and ammonia hydroxide was added drop by drop until the litmus paper changed, indicating alkaline pH decalcifying fluid was clear. 5 ml saturated ammonium oxalate solution was added when the decalcifying solution became turbid, indicating the presence of calcium within bone tissue so that the decalcifying solution was replaced with new solution, and the process was repeated every 30 minutes until the completion of the decalcification process. For EDTA, decalcifier physical testing was used in which the decalcification process was considered to have ended when the bone was easily penetrated through by a needle. The average total decalcified times were 7 hours and 30 minutes, 8 hours, and 120 hours for 5% nitric acid, 5% HCl, and 10% EDTA, respectively.

### 2.2. Microwave Oven Procedure

A house microwave oven (Midea Microwave 20L, 700W, Digital, EM720CFF) with an immovable rotating plate was used. A glass beaker containing 100 ml of distilled water was preheated for 5 seconds to warm up the magnetron. This was replaced by 100 ml of fresh distilled water and irradiated to maintain the temperature at around 41–43°C. This took 15 seconds. The glass beaker was allocated at various points in the oven while irradiating it to resolve the best location of the specimen during microwave decalcification since the microwave oven used had a constant timing but not a constant temperature. Three pieces of 5 mm-thick sections of bone biopsies were immersed in 250 ml Pyrex Squat Beakers containing 100 ml of 5% aqueous hydrochloric acid (HCl), 5% aqueous nitric acid (HNO_3_), and 10% EDTA. Then, they were transferred to the microwave oven, and the specimens were irradiated for ten cycles of ten seconds each (at 15-minute intervals) for a total time of 2–4 hours for acid decalcifiers (5% HNO_3_ and 5% HCl). The temperature of the decalcifying solution was maintained at around 41–43°C. The decalcifying solution and the endpoint of decalcification were checked, and the decalcifying solution was repeatedly changed until completion of decalcification. The end point of decalcification was checked using calcium oxalate and physical tests as aforementioned. The average total decalcified times were 3 hours and 45 minutes, 5 hours and 30 minutes, and 29 hours and 4 minutes for 5% nitric acid, 5% HCl, and 10% EDTA, respectively. After complete decalcification, the tissues were washed using distilled water and transferred into 0.3% ammonia solution for 5 minutes to neutralize the acid used.

### 2.3. Tissue Processing and Staining

The specimens were subjected to automatic tissue processing using the following protocols: the bone biopsies were placed in 10% formal saline for one hour. Then, 50% alcohol one hour, 70% alcohol one hour, 90% alcohol one hour, followed by 100% alcohol three changes each two hours each, xylene two changes, each for one and half hour, finally paraffin wax two changes each for two hours. The tissues were embedded in paraffin blocks and were sectioned to a thickness of 5-6 *μ*m using a rotary microtome. Sections were stained with Mayer's haematoxylin, as described by Mayer in 1903, and the counterstain in each was 1% eosin (HE) [14]. Gridley's stain was used for fungi demonstration as described by Gridley in 1953); after deparaffinization and rehydration, tissue sections were placed in 2% chromic acid for 30 minutes. They were then washed well with tap water, rinsed with distilled water, and then placed in Schiff's reagent for 20 minutes. They were then washed in running tap water for 10 minutes and rinsed with 70% ethanol and then with 95% ethanol. They were counterstained with Metanil Yellow for one minute and rinsed well with distilled water. Then, they were dehydrated in xylene and mounted in distyrene, a plasticizer, and xylene (DPX). Then, the sections were examined under a microscope [15]. Also, the Grocott hexamine-silver method for fungi as described by Grocott in 1955 was used in which sections were oxidized with 4% aqueous chromic acid for one hour, washed in water for a few seconds, treated with 1% sodium metabisulphite for one minute, washed in running tap water for 3 minutes, rinsed thoroughly in distilled water, placed in preheated working silver solution in a water bath at 60°C for 20 minutes, rinsed well in distilled water, washed with running tap water for 5 minutes, counterstained in working light green for 15 seconds, dehydrated and cleared in xylene and mounted in DPX [16], and finally examined under a microscope.

### 2.4. Evaluation of Results

Sections were evaluated by an expert histopathologist. The quality of the decalcification procedure and staining result were also assessed and evaluated by the rule of thumb, and the quality of decalcification was evaluated by the following criteria: the time of decalcification, the morphological preservation of tissue morphology, and *Madurella mycetomatis* fungi by HE; Gridley and Grocott methenamine-silver staining was graded from 1 to 4 (1: poor, 2: fair, 3: good, and 4: excellent) [17].

### 2.5. Statistical Analysis

Data analysis was performed using the SPSS program. One-way ANOVA was used to prove the effect of the three decalcifying solutions on the quantitative analysis of section quality and fungi preservation. The Kruskal–Wallis test was performed to determine if there was a significant difference between the solutions tested for each of the parameters evaluated along with the two experiments. Differences with *P* < 0.05 were interpreted as being statistically significant.

## 3. Results

**Figure 1:**
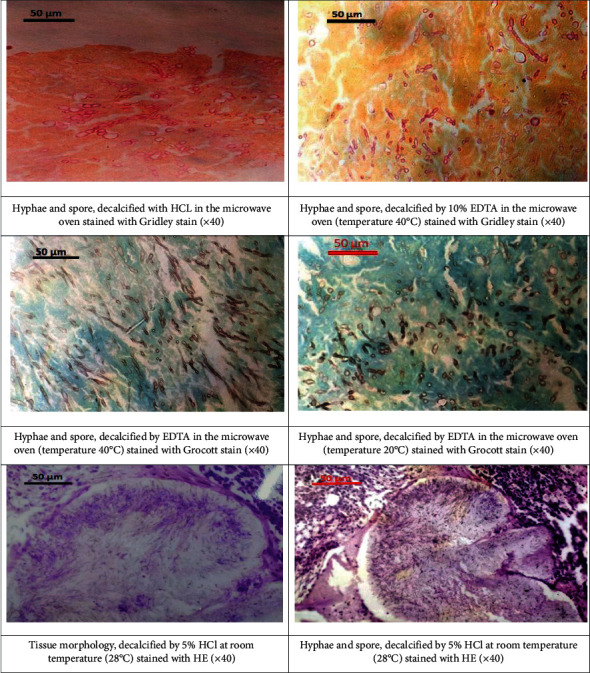
Demonstration of staining results using different decalcifying agents and conditions.

Fifty cases of *Madurella mycetomatis* with black grain were included in the study. The feet were the most affected anatomical region in 48 cases (96%) and two cases (4.0%) in the hand. With regard to time of decalcification in different experiments, among the three solutions tested, decalcification with 10% EDTA (pH 7.4) took the longest time for the conventional method (up to 120 hours compared to 29 hours with the microwave), and the use of an acid-decalcifying solution took the shortest time ranging from 8 hours to 3 hours for different decalcification methods. The quality of tissue morphology of different types of decalcifying solution using the decalcification microwave method compared with the conventional method with Mayer's haematoxylin and eosin staining with regard to nuclear and cytoplasmic appearance achieved variable results. Also, 10% EDTA-decalcified bone appeared to have a significantly superior result (*P* value using chi-square test: 0.023) compared with 5% HNO_3_ and 5% HCl. Excellent staining report was 43 (86%) versus 32 (64%), 42 (84%) versus 18 (36%), and 33 (66%) versus 1 (2%), respectively. The Grocott methenamine-silver stain method was used for demonstration of the Madurella mycetomatis causative agent concerning the morphology and brightness of fungi, and tissue sections decalcified by 10% EDTA, 5% HNO_3_, and 5% HCl using conventional and microwave methods showed significantly better fungal morphology (*P* value using chi-square test: 0.001) as follows: 33 (66%) versus 32 (64%), 33 (66%) versus 39 (78%), and 22 (44%) versus 31 (62%), respectively. The other fungal stain used for *Madurella mycetomatis* was Gridley stain concerning fungal morphology and staining quality of bones decalcified with 10% EDTA, 5% HNO_3_, and 5% HCl using the conventional and microwave methods which showed significantly better findings (*P* value using chi-square test: 0.003) as follows: 43 (86%) versus 41 (82%), 32 (64%) versus 34 (68%), and 23 (46%) versus 35 (70%), respectively. These results are summarized in Table 2.  Figure 1 shows the staining results using different decalcifying agents and conditions.

## 4. Discussion

The histopathological identification of the causative agent of Madurella mycetomatis is well established as it is the gold standard procedure; however, hard tissue and bone require special decalcification to preserve the tissue structure and causative agent morphology. The causative agents can be identified using haematoxylin and eosin (HE) and fungi special stains, so the bone requires a standard decalcification protocol that preserves tissue, causative agent morphology, and stain ability. Bone decalcification is a tedious technique. It requires weeks, and conservation of the tissue configuration depends on the excellence and speed of the decalcification procedure. A novel process using a microwave oven was realized to quicken the decalcification process [18]. The selection of the decalcifying agent and manner is basically determined by the earnestness of the method [18], and the possible uses of microwave energy in histological techniques were first documented by Mayers in 1970. This system of nonionizing emission method thought to speedup decalcification process . The molecular kinetics then cause the production of energy change, which lasts until the radiation stops [19]. In this study, decalcification times reported for microwave-enhanced decalcification and the conventional decalcification procedure were 29 and 120 hours with 10% EDTA (pH 7.4), three hours and seven hours with 5% nitric acid, and five and eight hours with 5% HCl, respectively. It is clear that the microwave decalcification method for bone tissue affected with mycetoma infection was significantly faster than the conventional method. Also, 5% nitric acid had faster decalcifying ability followed by 5% hydrochloric acid and then EDTA (pH 7.4) for both methods. Pitol et al. [18] used a home microwave cooker for removal of calcium of the rat bone by 8.5% EDTA solution and displayed a decrease of trial period from 45 days in the conventional process to 48 h in the microwave-assisted process. In this work, the 10% EDTA solution took 120 hours in the conventional method and 29 hours using microwaves to achieve complete decalcification of the bone tissue affected with mycetoma infection. The concentration of EDTA in our experiment was more than Pitol et al.'s [18] study. In addition to differences in size, thickness, and types of the bone, these may be possible explanations for the increases in time of decalcification in Pitol et al.'s [18] study that were reduced in our setting. Furthermore, decalcification of the bone affected with mycetoma infection with the conventional method using 5% nitric acid took seven hours, whereas the microwave oven method took three hours. Comparable outcomes were achieved by Balaton and Loget [20]. Furthermore, in this research, decalcification times were reduced from other studies. The decalcification times reported for conventional and microwave methods ranged from one day and four hours to 5 days and are considered low in comparison with other studies in the rodent bone, which have reported times between 2 and 7 days with acid-decalcifying solutions and 10% EDTA (pH 7.4), respectively [21]. Shibata and his group reported nearly similar decalcification times that varied from 1 day to 7 days with 10% HCl, 10% nitric acid, and 10% EDTA. However, they used acid decalcifiers with higher concentrations [22]. Uma et al. evaluated the effects of four different decalcifying fluids using microwave decalcification in the rat bone and reported 1 to 1.5 days for 5% nitric acid and 7% HCl/2% EDTA. However, 10% EDTA took 14 to 26 days according to the type of the bone [12]. In these studies, the bone type, nature, and size varied and differed from those analyzed here. The decalcifying procedure was a key reason for the superiority of the tissue section and precision of staining. Philipp et al. (2019) described that the decalcification method causes significant morphological changes [23] that alter the protein structure and affect staining ability of the tissue [24]. In our study, the bone was treated with 5% nitric acid with the routine manual method, and there was swelling in soft tissue and loss of nuclear and fungal staining compared with microwave-assisted decalcification. Acid-decalcifying agents usually disturb bone and soft tissue constancy. These special effects in 5% nitric acid are because of the period taken and the acidity of solution. Thus, faster decalcification will cause greater injury and larger effects in H&E and fungal special stain. Several fungi can be demonstrated with a histological section using histochemical stains such as Grocott's methenamine-silver (GMS) stain and/or Gridley (GS) stain [25]. Some fungi can be exactly demonstrated in tissue based on morphologic structures; however, the recognition efficacy tended to diminish after prolonged fixation and decalcification using conventional methods [26]. In this study, microwave-assisted decalcification gave superior staining compared with the conventional method for morphology using H&E and fungal special stain, where 10% EDTA was the solution that best preserved tissue structures and fungal staining ability despite taking a long time for decalcification.

## 5. Conclusion and Recommendations

Decalcification enhanced by the use of a microwave oven is a new technique for decalcification. This technique was investigated in bone tissue affected with Madurella mycetomatis infection using 10% EDTA, 5% nitric acid, and 5% hydrochloric acid as decalcifying fluids. This was compared with conventional decalcification at room temperatures to determine the speed of decalcification and tissue morphology using Mayer's haematoxylin and eosin stain for the bone structure and Grocott and Gridley stains for fungal morphology. Better results were achieved compared with the conventional method both in cell morphology and fungi spore and hyphae with decreased fungi brightness at room temperature. This finding may help to develop suitable protocols for the analyses of bone tissue affected with mycetoma infection.

## 6. Limitations of the Present Study

A larger sample size would have given more conclusive results. Also, the times taken for decalcification are likely to be different if different bone weights and bone samples other than hands are used since the decalcification time is dependent on the size and structural density of the hard tissue. Finally, since we have used a domestic microwave oven, our temperature recordings may have been only approximate.

## Figures and Tables

**Table 1:** The ingredients and preparation of different decalcifying agents.

Decalcifying agents	10% EDTA	5% nitric acid	5% hydrochloric acid
Preparation	100 g EDTA and 10 g sodium hydroxide	5 ml of nitric acid	5 ml of hydrochloric acid
Distilled water	Add to 1000 mL	Add to 95 mL	Add to 95 mL
pH	7.4	—	—

**Table 2:** Decalcifying solution scores as the measurement of decalcification time and morphological fungi preservation.

Decalcifying solutions	Decal/time RT/MW Hours/minutes	*M. mycetomatis* fungi morphological evaluation												Total score
		FSHE, RT/MW				FSG, RT/MW				FSGr, RT/MW				
		P	F	G	E	P	F	G	E	P	F	G	E	
10% EDTA	120 h/29 h: 4 min	3/1	5/2	10/4	32/43	1/5	1/7	15/6	33/32	1/0	1/0	5/9	43/41	50/50
5% nitric acid	7 h: 30min/3:45 min	2/3	2/3	37/2	9/42	5/3	4/4	8/4	33/39	1/3	2/4	15/9	32/34	50/50
5% HCl	8 h/5 h: 30 min	2/2	5/4	42/11	1/33	5/1	5/1	18/17	22/31	5/2	4/2	18/11	23/35	50/50
Chi-square test		*P* value: 0.023				*P* value: 0.001				*P* value: 0.03				

*Note.* Decal: decalcification. FSHE: fungi staining with haematoxylin and eosin. FSG: fungi staining with Gridley stain. FSGr: fungi staining with Grocott hexamine-silver stain. RT: room temperature. MW: microwave oven. P: poor. F: fair. G: good. E: excellent.

## Data Availability

The datasets generated during the current study are available from the corresponding author on reasonable request.

## Supplementary Materials

Supplementary Materials The materials and reagents used to support the findings of this study are included within the supplementary information.

## References

[1] Fahal A., Mahgoub E. S., Hassan A. M. E. L., Abdel-Rahman M. E. (2015). Mycetoma in the Sudan: an update from the mycetoma research Centre, university of Khartoum, Sudan. *PLoS Neglected Tropical Diseases*.

[2] Davis J. D., Stone P. A., McGarry J. J. (1999). Recurrent mycetoma of the foot. *The Journal of Foot and Ankle Surgery*.

[3] Schwartz E., Shpiro A. (2015). Madura foot or philoctetes foot?. *Israel Medical Association Journal*.

[4] Fahal A. H. (2017). Mycetoma. *Current Progress in Medical Mycology*.

[5] Abbas M., Scolding P. S., Yosif A. A. (2018). The disabling consequences of Mycetoma. *PLoS Neglected Tropical Diseases*.

[6] Hoog G. S., Adelmann D., Ahmed A. O. A., Belkum A. (2004). Phylogeny and typification of Madurella mycetomatis, with a comparison of other agents of eumycetoma. *Mycoses*.

[7] Findlay G. H., Vismer H. F., Botes D. P., Kruger H. (1980). Black grain mycetoma: studies on the pigment of Madurella mycetomi. *Mycopathologia*.

[8] Choube A., Astekar M., Choube A., Sapra G., Agarwal A., Rana A. (2018). Comparison of decalcifying agents and techniques for human dental tissues. *Biotechnic & Histochemistry*.

[9] Dermience M., Lognay G., Mathieu F., Goyens P. (2015). Effects of thirty elements on bone metabolism. *Journal of Trace Elements in Medicine and Biology*.

[10] Rohrer M. D., Schubert C. C. (1992). The cutting-grinding technique for histologic preparation of undecalcified bone and bone-anchored implants. *Oral Surgery, Oral Medicine, Oral Pathology*.

[11] Begum F., Zhu W., Namaka M. P., Frost E. E. (2010). A novel decalcification method for adult rodent bone for histological analysis of peripheral-central nervous system connections. *Journal of Neuroscience Methods*.

[12] Uma K., Chandavarkar V., Sangeetha R. (2014). Comparison of routine decalcification methods with microwave decalcification of bone and teeth. *Journal of Oral and Maxillofacial Pathology*.

[13] Srinivasyaiah A., Nitin P., Hegde U. (2016). Comparison of microwave versus conventional decalcification of teeth using three different decalcifying solutions. *Journal of Laboratory Physicians*.

[14] Drury R. (2007). Theory and practice of histological techniques. *Journal of Clinical Pathology*.

[15] Sangeetha J., Thangadurai D. (2013). *Laboratory Protocols in Fungal Biology*.

[16] Swisher B. L., Chandler F. W. (2016). Grocott-gomori methenamine silver method for detecting fungi: practical considerations. *Laboratory Medicine*.

[17] González-Chávez S. A., Pacheco-Tena C., Macías-Vázquez C. E., Luévano-Flores E. (2013). Assessment of different decalcifying protocols on Osteopontin and Osteocalcin immunostaining in whole bone specimens of arthritis rat model by confocal immunofluorescence. *International Journal of Clinical and Experimental Pathology*.

[18] Pitol D. L., Caetano F. H., Lunardi L. O. (2007). Microwave-induced fast decalcification of rat bone for electron microscopic analysis: an ultrastructural and cytochemical study. *Brazilian Dental Journal*.

[19] Choji T., Ngokere A., Ogenyi S., Kumbish P. (2015). Histo-architechtural evaluation of conventional versus two rapid microwave processing techniques. *British Biotechnology Journal*.

[20] Balaton A. J., Loget P. (1989). *Decalcification Acceleree Par Les Micro-Ondes*.

[21] Yamamoto-Fukuda T., Shibata Y., Hishikawa Y. (2000). Effects of various decalcification protocols on detection of DNA strand breaks by terminal dUTP nick end labelling. *Histochemical Journal*.

[22] Shibata Y., Fujita S., Takahashi H., Yamaguchi A., Koji T. (2000). Assessment of decalcifying protocols for detection of specific RNA by non-radioactive in situ hybridization in calcified tissues. *Histochemistry and Cell Biology*.

[23] Pieroh P., Ghadban C., Litvak L. (2019). The comparative analyses of decalcification procedures and methyl benzoate pre-treatment on tissue preservation and antigenicity in human acetabular labra. *Histology and Histopathology*.

[24] Meritet D. M., Spagnoli S. T., Fischer K. A., Lohr C. V. (2019). Evaluating the Effects of Various Decalcification Protocols on Immunohistochemical Staining in Zebrafish (*Danio rerio*). *Zebrafish*.

[25] Roden A. C., Schuetz A. N. (2017). Histopathology of fungal diseases of the lung. *Seminars in Diagnostic Pathology*.

[26] Sano M., Sugitani M., Ishige T. (2007). Supplemental utility of nested PCR for the pathological diagnosis of disseminated trichosporonosis. *Virchows Archiv*.

