# Supplementary material for: Comparison between Conventional Decalcification and a Microwave-Assisted Method in Bone Tissue Affected with Mycetoma
Source: Biochem Res Int. 2020 Aug 1;2020:6561980. doi: 10.1155/2020/6561980 (PMC7422918; doi:10.1155/2020/6561980)
Supplement: Supplementary Materials — The materials and reagents used to support the findings of this study are included within the supplementary information. [file 6561980.f1.docx]

**Materials and reagent used for this study:**

- Flasks for preparation of the reagents.
- Measuring cylinder.
- 100 slides + 100 cover glasses.
- Surgical gloves: sterilized by gamma radiation.
- Paraffin wax ceresin: melting point about (55-60)ºC.
- Oven.
- Microtome + Needle + forceps + floating water bath.
- House microwave oven (Midea Microwave 20L, 700W, Digital, EM720CFF)
- Coplin Jars.
- Microscope.
- Filter paper.
- Ice trays.
- Funnel
- Pencil and markers.
- Moulds for embedding.

**2/ Reagents:**

- Xylene AR C_6_H_4_ (CH_2_)_2_ M.W 106.17 . S.D Five-Chem LTD. MUMBAI 400.015.
- Haematoxylin Lin monohydrate C_16_H_14_×H_2_O M.W 302.29×H_2_O.S.D five -Chem LTD . MUMBAI 400.025.
- Eosin water soluble yellow shade the British drug house LTD England.
- D.P.X S.D five-Chem LTD.
- H_2_SO_4_.

**3/ Preparation of the reagents:**

**• 10% formal-saline:**

- Formalin 100ml

- Sodium chloride 8.5g

- Tap water 900ml

**• Egg albumin total:**

- White egg 2.5ml

- Glycerol 2.5ml

**• Mayer's Haematoxylin (100ml):**

- Haematoxylin 1g

- Potassium alum 25g

-citric acid 0.5g

- Sodium iodate 0.1g

- Chloral hydrate 25g

- D.W 500ml

**• Eosin (50ml):**

- Eosin 5g

- D.W 50ml

**• Gram's iodine (100ml):**

- Iodine crystals 0.34g

- Potassium iodine 0.67g

**• Silver solution:**

- 10% silver nitrate 20ml

- Ammonia drops

- D.W 20ml

**• safranin (50ml):**

- safranin 0.1g

- D.W 50ml

**• Ferric-ferricyanide solution(40ml):**

- 1% potassium ferricyanide 4ml

- 1% ferric chloride 30ml

- D.W 6ml

**• 4% Chromic acid (100ml):**

- Chromic acid 4g

- D.W 50ml

**• Aldehyde-fuchsin solution:**

- Basic fuchsin 0.5g

- 70% alcohol 100ml

- Paraldehyde 1ml

- Concentration HCL 1ml

**• Sulphrous acid rinse (50ml):**

- 10% aqueous potassium metabisulphate 2.5ml

- 0.1N HCL 2.5ml

- D.W 50ml

**• Metanil yellow solution (50ml):**

- 0.25% Metanil yellow 25ml

- 0.25% acetic acid 25ml

**• 1% aqueous periodic acid solution (50ml):**

- Periodic acid 0.5ml

- D.W 50ml
